# Supine position-related obstructive sleep apnea in children: insights from the Childhood Adenotonsillectomy Trial

**DOI:** 10.1007/s11325-025-03393-1

**Published:** 2025-06-30

**Authors:** Siyu Dai, Ming Yang, Chun Ting Au, Nobel Tsz Kin Yuen, Yuzheng Zhang, Agatha Tang, Michelle Wai Ling Yu, Albert Martin Li, Kate Ching Ching Chan

**Affiliations:** 1 Department of Paediatrics, The Chinese University of Hong Kong, Shatin, Hong Kong SAR China; 2 School of Clinical Medicine, Hangzhou Normal University, Hangzhou, China; 3 Department of Translational Medicine, Research Institute, The Hospital for Sick Children, Toronto, Canada; 4 Department of Health Technology Assessment, China National Health Development Research Center, Beijing, China; 5 Laboratory for Paediatric Respiratory Research, Li Ka Shing Institute of Health Sciences, Faculty of Medicine, The Chinese University of Hong Kong, Shatin, Hong Kong SAR China; 6 Hong Kong Hub of Paediatric Excellence, The Chinese University of Hong Kong, Kowloon, Hong Kong SAR China

**Keywords:** Obstructive sleep apnea, Children, Supine position, Adenotonsillectomy, Natural history

## Abstract

**Purpose:**

This study aimed to examine the effectiveness of adenotonsillectomy (AT) among pediatric obstructive sleep apnea (OSA) patients with and without supine position-related OSA (POSA) and explore the stability of this subtype over time.

**Methods:**

Data from the Childhood Adenotonsillectomy Trial (CHAT) were analyzed. Children with OSA were randomized to early AT (EAT) or watchful waiting with supportive care (WWSC). Polysomnographic and health outcomes were assessed at baseline and 7 months. POSA was defined as a supine obstructive apnea-hypopnea index (OAHI) ≥ 2× non-supine OAHI, with ≥ 30 min spent in each position.

**Results:**

Among 354 patients (mean age: 6.97 ± 1.39 years; male: 48.1%), 47.2% had POSA at baseline. Compared to non-POSA, children with POSA exhibited lower baseline OAHI [3.77 (2.48, 7.71) vs. 5.42 (3.03, 9.47) events/h, *p* = 0.006], longer rapid eye movement (REM) sleep in the supine position (*p* = 0.05), shorter REM sleep in non-supine position (*p* = 0.005), and fewer allergic conditions (37.7% vs. 48.4%, *p* = 0.05). Generalized linear models showed AHI reduction was associated with randomization grouping (*p* < 0.001) but not POSA status (*p* = 0.10). Our restricted cubic splines further supported this finding. Notably, in the WWSC group, POSA classification changed for half of the patients over 7 months, with changes in non-supine OAHI as a significant indicator.

**Conclusion:**

AT is effective in managing childhood OSA regardless of POSA status. The observed dynamic nature of POSA warrants future research into its pathophysiology and natural history.

**Clinical trial registration:**

Childhood Adenotonsillectomy Study for Children with OSAS (CHAT), Clinical Trial Identifier NCT00560859.

**Supplementary Information:**

The online version contains supplementary material available at 10.1007/s11325-025-03393-1.

## Introduction

Obstructive sleep apnea (OSA) is characterized by repeated episodes of partial or complete upper airway obstruction during sleep, leading to reduced airflow, recurrent desaturations and arousals [1]. The implications of OSA in children extend beyond disturbed sleep, encompassing a wide range of negative health and behavioral outcomes such as cardiovascular dysfunction, neurocognitive impairment and respiratory infection, which significantly affect the developmental trajectory of affected children [2–4]. OSA is a heterogeneous disease, thus recognizing subtypes within the broad diagnosis is critical for individualizing treatment and optimizing patient outcomes [5–8].

A disease subtype is typically characterized by a consistent natural history, specific clinical and physiologic features, and predictable therapeutic responses. In the context of OSA, supine position-related OSA (POSA), is a widely recognized subtype affecting approximately 20-60% of children diagnosed with OSA [9–12]. In the existing literature, pediatric POSA is primarily distinguished by respiratory events that predominantly occur when the patient sleeps in a supine position. Previous research on childhood POSA focuses on specific subgroups like obese children [10] and those with Down syndrome [11]. Older age, lower body mass index (BMI), milder sleep apnea and specific upper airway obstruction locations such as tongue base have been linked to this positional subtype [9–15], indicating unique pathophysiologic traits that necessitate tailored clinical management. Adenotonsillectomy (AT) is widely recognized as the first-line treatment option for pediatric OSA associated with adenotonsillar hypertrophy [16, 17]. However, the effectiveness of AT in children with POSA compared to their counterparts remains unknown despite its high prevalence in the pediatric population. This gap in knowledge underscores the necessity of dissecting the treatment effects by subtypes to inform treatment approaches [18].

Moreover, it remains unclear whether childhood POSA is a stable subtype [9–15], that earlier adult studies raised concerns about its potential instability and found weight gain as well as high apnea-hypopnea index (AHI) in the lateral position as indicators of the observed variations [19–21]. Nevertheless, the condition in children has not yet been evaluated. Assessing the natural progression of this common clinical subtype is vital to determine whether non-invasive alternative treatments, such as positional therapy (PT), which aims to prevent patients from sleeping on their backs, should be preferentially considered for this group of children [7, 22, 23]. The Childhood Adenotonsillectomy Trial (CHAT) is the largest randomized controlled clinical trial (RCT) comparing ‘early AT’ (EAT) with ‘watchful waiting with supportive care’ (WWSC) in managing childhood OSA. It is characterized by rigorous and comprehensive outcome assessments, which include polysomnographic parameters and comprehensive clinical outcomes [24–26]. In this study, we aimed to leverage the CHAT dataset to (1) examine the effectiveness of AT among pediatric OSA patients with and without POSA and (2) investigate the stability of POSA over a 7-month follow up.

## Methods

### Participants

In the CHAT study, eligible participants were children with polysomnography (PSG) confirmed OSA. Pediatric OSA was identified by an AHI ≥ 2 events/h or an obstructive apnea index (OAI) ≥ 1 event/h, and sleep position during PSG is determined using body position sensors and video recordings [25]. For this current research, inclusion was limited to children with at least 30 min of sleep recorded in both supine and non-supine positions, adhering to the widely recognized POSA diagnostic criteria of the Cartwright Classification [27] and previous pediatric research [9–15]. Moreover, in the stability analysis, included children were required to meet this sleep duration criterion at both baseline and endline PSG assessments.

The CHAT study obtained institutional review board approval and written informed consent. For this current secondary analysis, the data use agreement was secured from the National Sleep Research Resource (NSRR) as required.

### Study design

This secondary analysis of the CHAT dataset was conducted from June 2023 to October 2024. The CHAT study (NCT005600859) was a multicenter, single-blinded RCT evaluating if pediatric OSA patients without prolonged oxyhemoglobin desaturations assigned to EAT would exhibit greater improvements in outcome measures than children assigned to WWSC over a 7-month follow-up. Its major outcomes included PSG results, OSA symptoms, neurocognitive function, behavior change, and validated quality of life (QoL) measures at the study baseline and 7 months. Further details on its study design have been described in prior publications [24, 25]. In this current analysis, pediatric POSA was defined as obstructive apnea-hypopnea index (OAHI) supine ≥ 2×OAHI non-supine in line with the Cartwright Classification [27] and previous pediatric POSA research [9–15].

### Outcome measures

In the CHAT study, each child underwent a full-night PSG conducted by a trained technician prior to randomization. Certified technologists who were blinded to other CHAT study data performed the scoring employed the American Academy of Sleep Medicine (AASM) pediatric criteria. The PSG was re-administered after 7 months to evaluate outcomes. Assessments of OSA disease symptoms, behavior and QoL were conducted at the study baseline and again at the 7-month follow-up to assess changes over time. As for cognitive assessment, it was conducted separately using the Developmental Neuropsychological Assessment (NEPSY). Aligned with our research aim, AHI was prioritized as the primary outcome for its objectivity in measuring OSA severity, allowing comparison of AT effectiveness and analysis of subtype transitions.

Thus outcome variables analyzed in the current study encompassed: (1) objective sleep metrics and the severity of OSA, as indicated by AHI; (2) OSA symptoms, evaluated using the Pediatric Sleep Questionnaire-Sleep Related Breathing Disorders (PSQ-SRBD); (3) cognitive functioning, evaluated using the NEPSY; (4) behavioral scores, assessed by the Conners’ rating scale and the Behavior Rating Inventory of Executive Function (BRIEF); (5) QoL, assessed with the Pediatric QoL Questionnaire (PedQoL); and (6) frequency and associated factors of subtype transitions during the 7-month study period. Data from the WWSC group were employed to explore subtype stability and transitions.

### Statistical analysis

Baseline and end-line demographics, clinical characteristics and outcome measures obtained from the CHAT database were analyzed. Descriptive statistics included frequencies and percentages for categorical variables and means with standard deviations (± SD) or medians with interquartile ranges (IQR) for continuous variables. For group comparisons between children with POSA and their counterparts, categorical variables were analyzed using the Chi-square (χ2) tests, and continuous variables were examined using either independent-samples t-tests or Mann-Whitney U tests as appropriate. To assess the impact of POSA status on treatment outcomes, multiple generalized linear models (GLM) and restricted cubic splines (RCS) analyses were utilized to robustly explore potential relationships and adjust for potential non-linearity. To explore the natural course of POSA in the WWSC group, children with unresolved OSA (AHI ≥ 2 events/h or OAHI ≥ 1 event/h) at the trial end-line were included. Regarding group comparisons and identification of indicators for POSA subtype transition, t-tests or Mann-Whitney U tests were performed as appropriate.

For sample size calculation, due to the lack of prior studies on the therapeutic effects of AT between POSA and non-POSA patients, a medium effect size and a POSA prevalence of 40% was assumed. Using G*Power 3.1 for a GLM (α = 0.05, power = 80%, Cohen’s f² = 0.02), the required sample size was calculated to be 315 participants, accounting for a 20% attrition rate and assuming equal allocation between treatment modalities (EAT vs. WWSC). This ensures sufficient statistical power to detect multiple group differences. All analyses in the current study were performed based on the intention-to-treat principle and using R version 4.4.0 and SPSS version 23.0, with *p* ≤ 0.05 considered as significant.

## Results

### Characteristics of the children included

A total of 354 participants (mean age: 6.97 ± 1.39 years; male: 48.1%) were included in this analysis (Table 1). About one-third of the participants were classified as having obesity, and the severity of OSA was categorized as follows: 54.2% mild OSA (1 event/h ≤ OAHI < 5 events/h), 26.0% moderate (5 events/h ≤ OAHI < 10 events/h) and 19.8% severe (OAHI ≥ 10 events/h). The prevalence of POSA was found to be 47.2% within the current cohort at the study baseline. Upon analyzing the demographic and clinical variables, no significant differences were observed in age, gender or obesity status between the POSA and non-POSA groups. Notably, children with POSA had significantly lower OAHI compared to their counterparts [3.77 (2.48, 7.71) events/h vs. 5.42 (3.03, 9.47) events/h, *p* = 0.006]. Children with POSA also had longer average REM sleep in the supine position (31.16 min vs. 26.06 min, *p* = 0.05), shorter REM sleep in the non-supine position (52.51 min vs. 61.41 min, *p* = 0.005) compared to non-POSA ones, which likely reflect the interaction between body position and sleep stages. Moreover, children with POSA also shown to with fewer allergic conditions (37.7% vs. 48.4%, *p* = 0.05) compared to their counterparts.

**Table 1:** Baseline demographic and clinical characteristics (*N* = 354)

Variables	POSA (*N* = 167)	Non-POSA (*N* = 187)	*P* ^†^
Age, years	7.1 ± 1.4	6.8 ± 1.4	0.06
Female, N (%)	96 (57.5%)	88 (47.1%)	0.06
Race, N (%)			
White	66 (39.5%)	60 (32.1%)	0.15
Black	82 (49.1%)	111 (59.4%)	
Others	19 (11.4%)	16 (8.6%)	
Family annul income before taxes, N (%)			
<$20,000	49 (29.3%)	49 (26.2%)	0.77
$20,000 to $40,000	39 (23.4%)	48 (25.7%)	
Others	79 (47.3%)	90 (48.1%)	
Obesity, N (%)	60 (32.1%)	61 (36.5%)	0.43
BMI z-score	0.96 ± 1.27	0.83 ± 1.36	0.38
Neck circumstance, cm	27.85 ± 2.85	27.84 ± 3.00	0.98
Waist circumstance, cm	63.30 ± 13.02	61.94 ± 13.02	0.33
RCT grouping, N (%)			
Early AT	80 (47.9%)	94 (50.3%)	0.67
WWSC	87 (52.1%)	93 (49.7%)	
Premature born, N (%)	33 (19.8.%)	27 (14.4%)	0.18
Pregnancy maternal smoke, N (%)	26 (15.6%)	25 (13.4%)	0.35
Primary caregiver daily smoke, N (%)	34 (20.4%)	39 (20.9%)	0.91
Child any allergy, N (%)	63 (37.7%)	90 (48.4%)	0.05
Jogging or running, days/wk.	1.8 ± 2.3	2.3 ± 2.7	0.08
Tonsillar size^*^, N (%)			
51-100%	41 (24.7%)	53 (28.5%)	0.47
0–50%	125 (75.3%)	133 (71.5%)	
Age started snoring	4.7 ± 11.1	3.6 ± 7.7	0.36
Parental enlarged tonsils or adenoids, N (%)	57 (34.1%)	57 (30.5%)	0.46
OSA severity, N (%)			
Mild (1 event/h ≤ OAHI < 5 events/h)	104 (62.3%)	88 (47.1%)	0.02
Moderate (5 events/h ≤ OAHI < 10 events/h)	36 (21.6%)	56 (29.9%)	
Severe (OAHI ≥ 10 events/h)	27 (16.2%)	43 (23.0%)	
Total sleep duration, minutes	453.34 ± 55.07	463.03 ± 49.73	0.08
Percentage of sleep in supine position	39.70 ± 22.71	40.08 ± 18.76	0.85
Percentage of REM sleep	18.10 ± 4.27	18.58 ± 4.32	0.30
Total REM sleep in supine position, minutes	31.16 ± 25.81	26.06 ± 23.43	0.05
Total REM sleep in non-supine position, minutes	52.51 ± 30.80	61.41 ± 27.81	0.005
Total NREM sleep in supine position, minutes	148.01 ± 85.77	159.42 ± 77.17	0.19
Total NREM sleep in non-supine position, minutes	222.28 ± 90.16	216.92 ± 76.26	0.55
OAHI (no. of events/h), Median (Range)	3.77 (2.48 to 7.71)	5.42 (3.03 to 9.47)	0.006
OAHI REM (no. of events/h), Median (Range)	7.02 (2.14 to 17.76)	10.75 (4.14 to 24.18)	0.003
OAHI NREM (no. of events/h), Median (Range)	2.99 (1.92 to 5.08)	3.61 (2.17 to 6.39)	0.05

Data were presented as mean ± SD unless otherwise indicated

^*^Percentage of tonsils outside of the fossa and occupy of the oropharyngeal width at the study baseline

^†^P values were calculated using chi-square tests, independent-sample t-test or Mann-Whitney U test as appropriate

Definition of abbreviations: POSA = positional obstructive sleep apnea; RCT = randomized controlled trial; AT = adenotonsillectomy; WWSC = watchful waiting plus supportive care; OSA = obstructive sleep apnea; REM = rapid eye movement; NREM = non-rapid eye movement; OAHI = the obstructive apnea–hypopnea index; IQR = the interquartile range

OAHI: (Obstructive apneas with no oxygen desaturation threshold used and with or without arousal + hypopneas with > 50% flow reduction and > = 3% oxygen desaturation or with arousal) / hour of sleep from type I polysomnography

The definition of hypopnea events is consistent with the American Academy of Sleep Medicine (AASM) 2007 Manual (pediatric)

### AT effectiveness by POSA status

**Fig. 1:**
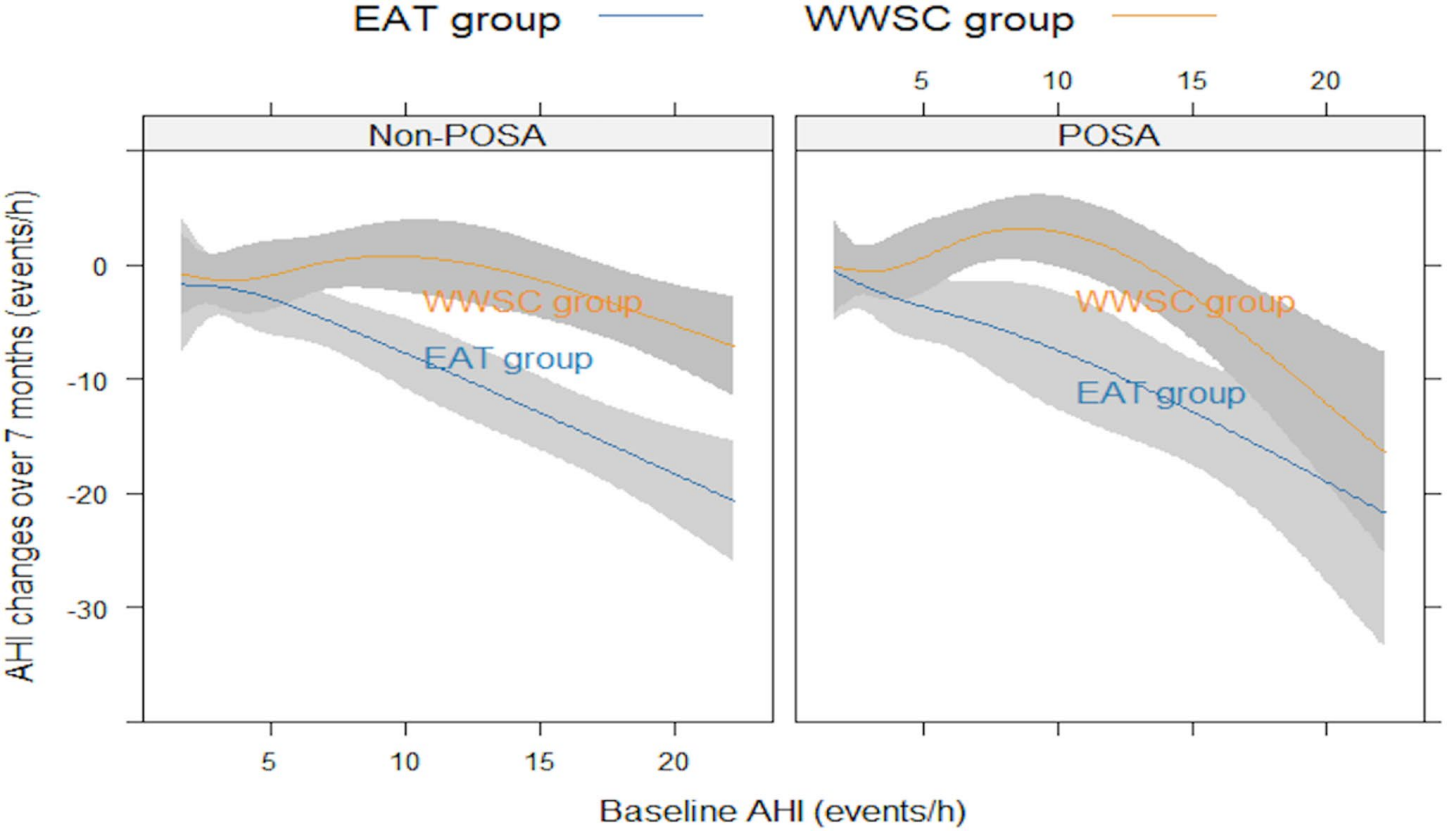
Comparative analyses of AHI reduction over 7 months in children by POSA status. Notes: the restricted cubic spline (RCS) with 4 knots was used to assess the impacts of POSA status on AT treatment effectiveness and its interaction with RCT grouping in children; The shadow represents 95% confidence intervals

Regarding surgical treatment effectiveness, our stratified comparison by RCT group showed that in the EAT group, children with POSA experienced a significantly smaller reduction in AHI compared to children with non-POSA [-2.61 (-5.36, -1.81) events/h vs. -4.93 (-8.75, -2.22) events/h, *p* = 0.02]. This difference was not observed in the WWSC group (Table 2). Moreover, another analysis stratified by POSA status (Supplementary Table 1) indicated that children with POSA experienced significantly smaller reduction in AHI compared to their counterparts at the 7-month endline visit regardless of the RCT allocation [-2.18 (-4.81, -0.71) events/h vs. -3.17 (-6.97, -1.11) events/h, *p* = 0.03]. EAT was significantly more effective in reducing AHI (*p* ≤ 0.001) among both POSA and non-POSA children compared to WWSC. Importantly, according to our GLM models, the decrease of AHI was only significantly associated with RCT grouping (EAT vs. WWSC) (*p* < 0.001) but not POSA status (yes vs. no) (*p* = 0.10). Results remained similar with different panels of adjustment, which included covariates such as children’s age, gender, BMI z-score, race and study centers (Table 3). No interaction was identified between RCT grouping and POSA in the GLM models. Our RCS further supported that EAT was generally effective in reducing AHI across patients with varying baseline AHI regardless of their POSA status, and the effectiveness was more obvious in children with more severe baseline disease (Fig. 1).

**Table 2:** AT effectiveness in outcome changes by RCT grouping and POSA status (*N* = 354)

Variables	Early AT (*N* = 174)				WWSC (*N* = 180)				*P* ^‡^
Trial outcome changes	**All**	POSA (*N* = 80)	Non-POSA (*N* = 94)	P^*^	All	POSA (*N* = 86)	Non-POSA (*N* = 94)	P^†^	
AHI (no. of events/h), Median (IQR range)	-3.81 (-7.17 to -1.89)	-2.61 (-5.36 to -1.81)	-4.93 (-8.75 to -2.22)	0.02	-1.99 (-4.39 to 0.42)	-1.66 (-4.10 to 0.69)	-2.21 (-5.01 to 0.40)	0.31	< 0.001
AHI supine (no. of events/h), Median (IQR range)	-5.32 (-9.87 to -2.31)	-6.03 (-14.32 to -3.21)	-3.46 (-8.71 to -1.66)	0.003	-2.48 (-6.96 to 1.33)	-3.63 (-8.79 to -0.67)	-1.18 (-4.51 to 3.25)	0.002	< 0.001
AHI non-supine (no. of events/h), Median (IQR range)	-2.50 (-6.01 to -0.76)	-0.96 (-1.89 to -0.20)	-5.02 (-8.78 to -2.61)	< 0.001	-0.90 (-2.88 to 0.55)	-0.36 (-1.00 to 1.89)	-1.75 (-5.03 to -0.33)	< 0.001	< 0.001
PSQ-SRBD score^&^	-0.27 ± 0.19	-0.28 ± 0.20	-0.27 ± 0.18	0.81	-0.03 ± 0.19	-0.04 ± 0.18	-0.02 ± 0.19	0.58	< 0.001
NEPSY attention and executive-function score^#^	7.75 ± 14.36	7.00 ± 15.43	8.44 ± 13.37	0.54	4.97 ± 14.01	5.69 ± 13.93	4.29 ± 14.14	0.53	0.09
Conners’ Rating Scale score^^^									
Caregiver rating	-2.93 ± 10.51	-3.97 ± 12.32	-2.00 ± 8.58	0.26	-0.31 ± 9.60	-0.79 ± 9.07	0.15 ± 10.12	0.54	0.02
Teacher rating	-5.01 ± 13.11	-4.71 ± 14.05	-5.22 ± 12.57	0.86	-1.31 ± 10.56	-0.83 ± 11.93	-1.80 ± 9.07	0.68	0.05
BRIEF score^@^									
Caregiver rating	-3.54 ± 8.39	-4.00 ± 9.34	-3.13 ± 7.45	0.53	0.67 ± 9.00	0.55 ± 9.13	0.78 ± 8.94	0.88	< 0.001
Teacher rating	-3.90 ± 12.09	-3.92 ± 13.06	-3.90 ± 11.45	0.99	-0.50 ± 11.97	0.63 ± 10.88	-1.69 ± 13.06	0.39	0.07
PedsQL score^**^	3.72 ± 14.89	5.18 ± 14.32	2.37 ± 15.37	0.25	2.91 ± 17.04	2.43 ± 17.68	3.38 ± 16.49	0.73	0.66
Persistent OSA, N (%)	60 (34.5%)	27 (33.8%)	33 (35.1%)	0.85	107 (59.4%)	53 (61.6%)	54 (57.4%)	0.57	< 0.001

Data were presented as mean ± SD unless otherwise indicated

Changes of the outcome measures: Endline values at the 7-month’s follow up - Baseline values

^*^Group comparison between the POSA and non-POSA children within the RCT’s early AT group

^†^Group comparison between the POSA and non-POSA children within the RCT’s WWSC group

^‡^Group comparison between the early AT and WWSC group among the included children

Definition of abbreviations: POSA = positional obstructive sleep apnea; RCT = randomized controlled trial; AT = adenotonsillectomy; WWSC = watchful waiting plus supportive care; AHI = the apnea–hypopnea index; IQR = the interquartile range

^&^The Pediatric Sleep Questionnaire sleep-related breathing disorder scale (PSQ-SRBD) has scores that vary from 0 to 1, where higher scores reflect more severe symptoms

^#^In the attention and executive-function domain of the Developmental Neuropsychological Assessment (NEPSY), scores range from 50 to 150, with higher scores representing better cognitive functioning

^^^ Scores on the Conners’ Caregiver Rating Scale Revised: Long Version Global Index, which includes the Restless–Impulsive and Emotional Lability factor sets, span from 38 to 90, with higher scores denoting poorer functioning

The Conners’ Teacher Rating Scale Revised also measures similar aspects with scores ranging from 40 to 90, where higher scores indicate poorer functioning

^@^On the Behavior Rating Inventory of Executive Function (BRIEF) Global Executive Composite, which includes summary measures of behavioral regulation and metacognition, higher scores suggest worse executive functioning. Scores range from 28 to 101 for caregiver ratings and from 37 to 131 for teacher ratings

**The Pediatric Quality of Life Inventory (PedsQL) scores between 0 and 100, with higher scores indicating a better quality of life

Persistent OSA: post-study AHI ≥ 2 events/h

**Table 3:** AT effectiveness in AHI reduction by RCT grouping and POSA status- GLM models (*N* = 354)

Variables	B	95% CI	Wald χ²	*P*
Model 1^&^				
AT Surgery (EAT vs. WWSC)	-4.291	-6.994 to -1.589	9.69	< 0.001
POSA (Yes vs. No)	1.544	-1.047 to 4.136	1.37	0.103
POSA * AT Surgery^‡^	0.038	-3.718 to 3.793	0.001	0.984
Model 2^*^				
AT Surgery (EAT vs. WWSC)	-4.369	-7.166 to -1.572	9.373	0.002
POSA (Yes vs. No)	1.529	-1.124 to 4.182	1.275	0.259
POSA * AT Surgery^‡^	0.104	-3.763 to 3.971	0.003	0.958
Model 3#				
AT Surgery (EAT vs. WWSC)	-4.576	-7.120 to -2.032	12.425	< 0.001
POSA (Yes vs. No)	0.640	-1.803 to 3.084	0.264	0.607
POSA * AT Surgery^‡^	-0.242	-3.756 to 3.272	0.018	0.893

For AT Surgery (EAT vs. WWSC), WWSC is the reference group; for POSA (Yes vs. No), No is the reference group

Definition of abbreviations: POSA = positional obstructive sleep apnea; RCT = randomized controlled trial; EAT = early adenotonsillectomy; WWSC = watchful waiting plus supportive care; AHI = the apnea–hypopnea index

AHI reduction: Endline values - Baseline values

^&^Model 1 is without covariates’ adjustment

*Model 2 adjusted for children’s age, gender and BMI z-score

^#^Model 3 adjusted for children’s age, gender and BMI z-score, and additionally adjusted for children’s race, RCT sleep study center and baseline AHI

^‡^The interaction term of the GLM model

Regarding other outcomes, including disease symptoms, neurocognitive function, and QoL, the effectiveness of AT was not affected by the POSA subtypes, with no significant interaction found between the treatment arm and the POSA status (Table 4).

**Table 4:** AT effectiveness in OSA symptom alleviation, cognition, behavior and QoL by RCT grouping and POSA status- GLM models (*N* = 354)

Variables	B	95% CI	Wald χ²	*P*
PSQ-SRBDscore&				
AT Surgery (EAT vs. WWSC)	-0.238	-0.297 to -0.178	61.163	< 0.001
POSA (Yes vs. No)	-0.017	-0.074 to 0.041	0.317	0.571
POSA * AT Surgery	0.009	-0.074 to 0.092	0.047	0.828
NEPSY#				
AT Surgery (EAT vs. WWSC)	1.308	-3.231 to 5.846	0.319	0.089
POSA (Yes vs. No)	1.403	-2.960 to 5.766	0.397	0.992
POSA * AT Surgery	-2.839	-9.135 to 3.456	0.781	0.377
Conners’ Caregiver rating^				
AT Surgery (EAT vs. WWSC)	-3.176	-6.411 to 0.058	3.704	0.020
POSA (Yes vs. No)	-0.941	-4.037 to 2.154	0.355	0.202
POSA * AT Surgery	-1.03	-5.507 to 3.447	0.203	0.652
Conners’ Teacher rating^				
AT Surgery (EAT vs. WWSC)	-3.873	-9.220 to 1.474	2.015	0.049
POSA (Yes vs. No)	0.972	-4.117 to 6.060	0.140	0.687
POSA * AT Surgery	-0.453	-7.709 to 6.804	0.015	0.903
BRIEF Caregiver rating@				
AT Surgery (EAT vs. WWSC)	-4.553	-7.359 to -1.747	10.113	< 0.001
POSA (Yes vs. No)	-0.225	-2.940 to 2.490	0.026	0.581
POSA * AT Surgery	-0.647	-4.538 to 3.245	0.106	0.745
BRIEF teacher rating@				
AT Surgery (EAT vs. WWSC)	-4.551	-9.892 to 0.791	2.788	0.071
POSA (Yes vs. No)	2.326	-2.905 to 7.557	0.760	0.538
POSA * AT Surgery	-2.347	-9.692 to 4.998	0.392	0.531
PedsQL score**				
AT Surgery (EAT vs. WWSC)	2.755	-2.401 to 7.910	1.097	0.635
POSA (Yes vs. No)	-0.956	-5.991 to 4.078	0.139	0.614
POSA * AT Surgery	3.765	-3.429 to 10.959	1.052	0.305

For AT Surgery (EAT vs. WWSC), WWSC is the reference group; for POSA (Yes vs. No), No is the reference group

Change of the outcome measures: Endline values - Baseline values

Definition of abbreviations: POSA = positional obstructive sleep apnea; RCT = randomized controlled trial; EAT = early adenotonsillectomy; WWSC = watchful waiting plus supportive care

^&^The Pediatric Sleep Questionnaire sleep-related breathing disorder scale (PSQ-SRBD) has scores that vary from 0 to 1, where higher scores reflect more severe symptoms

^#^In the attention and executive-function domain of the Developmental Neuropsychological Assessment (NEPSY), scores range from 50 to 150, with higher scores representing better cognitive functioning

^^^ Scores on the Conners’ Caregiver Rating Scale Revised: Long Version Global Index, which includes the Restless–Impulsive and Emotional Lability factor sets, span from 38 to 90, with higher scores denoting poorer functioning. The Conners’ Teacher Rating Scale Revised also measures similar aspects with scores ranging from 40 to 90, where higher scores indicate poorer functioning

^@^On the Behavior Rating Inventory of Executive Function (BRIEF) Global Executive Composite, which includes summary measures of behavioral regulation and metacognition, higher scores suggest worse executive functioning. Scores range from 28 to 101 for caregiver ratings and from 37 to 131 for teacher ratings

**The Pediatric Quality of Life Inventory (PedsQL) scores between 0 and 100, with higher scores indicating a better quality of life

### Stability of POSA over time

The CHAT project provided an opportunity to explore the natural course of POSA in the control group. Among WWSC children classified as POSA at baseline, 48.1% transitioned to non-POSA at the 7-month visit; 47.4% of those initially classified as non-POSA transitioned to POSA by the end (Table 5). In exploring potential indicators, children with non-POSA at baseline who transitioned to POSA at follow-up had a higher baseline percentage of REM sleep (19.38% vs. 17.02%, *p* = 0.04) compared to those who remained non-POSA (Table 6). Significant differences were found regarding changes in non-supine OAHI over the 7-month trial between those who transitioned and those did not (Table 7). For children with POSA at baseline, those who transitioned to non-POSA experienced an increase in the non-supine OAHI [2.03 (0.28, 3.88) vs. -0.68 (-2.09, 0.20), *p* < 0.001] compared to those who remained POSA. In contrast, among children with non-POSA at baseline, those who transitioned to POSA had a larger reduction in the non-supine OAHI [-2.24 (-9.77, -1.34) vs. -0.07 (-4.56, 2.83), *p* = 0.007]. No other factors were found to be significantly different between groups.

**Table 5:** The transition of POSA status in the WWSC group (*N* = 111)

Transition from baseline to follow-up	WWSC
POSA at baseline (*N* = 54)	
POSA to non-POSA	26 (48.1%)
POSA to POSA	28 (51.9%)
Non-POSA at baseline (*N* = 57)	
Non-POSA to POSA	27 (47.4%)
Non-POSA to Non-POSA	30 (52.6%)

Children with unresolved OSA (AHI ≥ 2 events/h or OAHI ≥ 1 event/h) at the trial endline were included in the analysis

POSA were determined by (1) an OAHI in the supine position ≥ 2× OAHI in the non-supine position; (2) each participant was required that spent at least 30 min in each sleeping positions (supine and non-supine) both at the study baseline and endline in the current analysis; (3) AHI ≥ 2 events/h OR an OAHI ≥ 1 events/h

Transition stands for POSA status change from the study baseline to the 7-month endline

**Table 6:** Comparisons of baseline characteristics by POSA subtype transitions in WWSC group (*N* = 111)

Variables	POSA at baseline (*N* = 54)			Non-POSA at baseline (*N* = 57)		
	POSA to Non-POSA (*N* = 26)	POSA to POSA (*N* = 28)	P	Non-POSA to POSA (*N* = 27)	Non-POSA to Non-POSA (*N* = 30)	P
Age, years	6.81 ± 1.26	7.06 ± 1.33	0.47	6.87 ± 1.39	6.92 ± 1.40	0.90
Female, N (%)	13 (50.0%)	17 (60.7%)	0.58	13 (48.1%)	14 (46.7%)	1.00
Race, N (%)			0.63			0.50
White	10 (38.5%)	12 (42.9%)		12 (44.4%)	9 (30.0%)	
Black	14 (53.8%)	12 (42.9%)		13 (48.1%)	19 (63.3%)	
Others	2 (7.7%)	4 (14.3%)		2 (7.4%)	2 (6.7%)	
Obesity, N (%)	10 (38.5%)	12 (42.9%)	0.79	13 (43.3%)	8 (29.6%)	0.41
Weight, KG	29.67 ± 10.33	32.69 ± 15.53	0.41	29.57 ± 11.55	31.23 ± 11.15	0.58
Z-score for weight-for-age	1.07 ± 1.39	1.10 ± 1.31	0.92	0.94 ± 1.21	1.31 ± 1.01	0.22
BMI z-score	1.11 ± 1.31	1.11 ± 1.14	0.99	0.83 ± 1.28	1.32 ± 0.91	0.11
Neck circumstance, cm	27.72 ± 2.19	28.11 ± 3.38	0.61	27.91 ± 2.87	28.36 ± 2.76	0.55
Waist circumstance, cm	61.80 ± 10.42	65.38 ± 16.39	0.34	61.66 ± 12.86	64.73 ± 13.65	0.39
Premature born, N (%)	3 (11.5%)	7 (25.0%)	0.28	1 (3.3%)	4 (14.8%)	0.18
Pregnancy maternal smoke, N (%)	3 (11.5%)	4 (14.3%)	0.32	7 (23.3%)	7 (25.9%)	1.00
Caregiver daily smoke, N (%)	6 (23.1%)	7 (25.0%)	1.00	6 (22.2%)	8 (26.7%)	0.77
Child any allergy, N (%)	12 (46.2%)	12 (42.9%)	1.00	7 (25.9%)	15 (50.0%)	0.10
Jogging or running, days/wk.	1.46 ± 1.94	1.89 ± 2.33	0.47	1.96 ± 2.58	2.37 ± 2.81	0.58
Tonsillar size^*^, N (%)			0.76			0.59
0–50%	8 (32.0%)	7 (25.0%)		10 (33.3%)	11 (40.7%)	
51-100%	17 (68.0%)	21 (75.0%)		20 (66.7%)	16 (49.3%)	
Parental enlarged tonsils or adenoids, (%)	9 (37.5%)	11 (40.7%)	0.37	8 (27.6%)	6 (23.1%)	0.93
Age started snoring	3.05 ± 1.77	2.12 ± 1.54	0.10	2.89 ± 1.52	2.78 ± 1.83	0.83
OSA severity, N (%)			0.68			0.23
Mild (1 ≤ OAHI ≤ 5 events/h)	12 (46.2%)	16 (57.1%)		9 (30.0%)	14 (51.9%)	
Moderate (5 < OAHI ≤ 10events/h)	8 (30.8%)	6 (21.4%)		10 (33.3%)	7 (25.9%)	
Severe (OAHI > 10 events/h)	6 (23.1%)	6 (21.4%)		11 (36.7%)	6 (22.2%)	
Total sleep duration, minutes	447.77 ± 51.92	433.39 ± 59.20	0.35	469.48 ± 37.27	452.27 ± 65.89	0.23
Percentage of REM sleep	17.34 ± 5.26	17.96 ± 4.57	0.65	19.38 ± 3.50	17.02 ± 4.78	0.04
Total duration of REM sleep in supine position, minutes	34.73 ± 19.85	29.86 ± 29.36	0.48	27.07 ± 22.38	22.10 ± 24.53	0.43
Total duration of REM sleep in non-supine position, minutes	44.81 ± 34.97	49.82 ± 29.20	0.57	64.63 ± 25.22	57.37 ± 30.64	0.34
Total duration of NREM sleep in supine position, minutes	166.88 ± 87.96	147.50 ± 84.42	0.41	173.33 ± 85.07	149.73 ± 78.25	0.28
Total duration of NREM sleep in non-supine position, minutes	201.88 ± 95.43	206.71 ± 84.57	0.84	205.11 ± 90.59	224.00 ± 75.30	0.39
Percentage duration of sleep in supine position	48.68 ± 26.02	39.84 ± 24.08	0.20	43.53 ± 18.94	45.53 ± 19.47	0.74
Baseline OAHI (no. of events/h), Median (IQR range)	6.04 (2.29 to 9.61)	4.15 (3.35 to 8.31)	0.87	4.61 (2.50 to 9.42)	9.21 (3.77 to 12.36)	0.13
OAHI supine, Median (IQR range)	10.21 (4.79 to 15.65)	7.95 (4.77 to 21.67)	0.80	4.09 (2.24 to 8.29)	6.85 (2.73 to 12.56)	0.26
OAHI non-supine, Median (IQR range)	1.98 (1.08 to 4.16)	2.16 (1.06 to 4.82)	0.90	4.62 (2.09 to 12.20)	7.77 (3.79 to 13.99)	0.17
OAHI supine/OAHI non-supine	3.84 (2.71 to 7.24)	3.33 (2.63 to 9.29)	0.98	0.91 (0.59 to 1.51)	0.95 (0.55 to 1.27)	0.84

Children with unresolved OSA (AHI ≥ 2 events/h or OAHI ≥ 1 event/h) at the trial endline were further included in the analysis

Data were presented as mean ± SD unless otherwise indicated

^*^Percentage of tonsils outside of the fossa and occupy of the oropharyngeal width at the study baseline

OAHI: (Obstructive apneas with no oxygen desaturation threshold used and with or without arousal + hypopneas with > 50% flow reduction and > = 3% oxygen desaturation or with arousal) / hour of sleep from type I polysomnography

The definition of hypopnea events is consistent with the American Academy of Sleep Medicine (AASM) 2007 Manual (pediatric)

**Table 7:** Comparisons of the changes in patient characteristics by POSA subtype transition in WWSC group (*N* = 111)

Variables	POSA at baseline (*N* = 54)			Non-POSA at baseline (*N* = 57)		
	POSA to Non-POSA (*N* = 26)	POSA to POSA (*N* = 28)	P	Non-POSA to POSA (*N* = 27)	Non-POSA to Non-POSA (*N* = 30)	P
Weight change, KG	3.14 ± 2.20	2.58 ± 1.88	0.33	2.52 ± 1.54	3.43 ± 2.26	0.08
Z-score for weight-for-age change	0.07 ± 0.26	0.06 ± 0.23	0.96	0.07 ± 0.27	0.14 ± 0.24	0.33
BMI z-score change	0.08 ± 0.27	0.01 ± 0.23	0.67	0.06 ± 0.87	0.24 ± 0.32	0.28
Neck circumstance change, cm	0.79 ± 1.12	0.68 ± 1.57	0.78	0.75 ± 0.82	0.41 ± 0.88	0.14
Waist circumstance change, cm	3.80 ± 5.33	1.85 ± 4.01	0.15	1.62 ± 2.80	2.81 ± 4.13	0.22
Change in total sleep duration, minutes	18.54 ± 60.97	15.21 ± 71.08	0.86	-2.37 ± 44.44	-10.43 ± 57.37	0.56
Percentage of REM sleep	-0.16 ± 5.78	0.22 ± 4.69	0.79	-0.03 ± 4.78	-0.33 ± 4.93	0.82
Total duration of REM sleep in supine position, minutes	-0.85 ± 30.30	-5.43 ± 25.79	0.55	9.19 ± 29.03	-1.03 ± 30.85	0.21
Total duration of REM sleep in non-supine position, minutes	2.92 ± 34.74	7.43 ± 31.98	0.62	-9.63 ± 37.55	-3.67 ± 33.63	0.53
Total duration of NREM sleep in supine position, minutes	24.46 ± 100.20	8.82 ± 107.22	0.58	-6.30 ± 112.81	29.33 ± 98.29	0.21
Total duration of NREM sleep in non-supine position, minutes	-7.96 ± 87.02	4.50 ± 90.28	0.61	4.26 ± 118.72	-35.33 ± 92.18	0.16
Percentage duration of sleep in supine position	2.84 ± 25.17	-0.70 ± 24.59	0.60	0.81 ± 27.96	7.66 ± 25.43	0.34
Change in OAHI	-1.40 (-3.64 to 0.63)	-0.90 (-3.96 to 1.22)	0.64	-1.44 (-6.81 to 4.35)	-1.12 (-3.42 to 1.73)	0.90
Change in OAHI supine, Median (IQR range)	-3.63 (-8.34 to -1.46)	-2.21 (-10.42 to 1.33)	0.35	0.58 (-2.25 to 7.46)	-1.18 (-7.02 to 5.69)	0.29
Change in OAHI non-supine, Median (IQR range)	2.03 (0.28 to 3.88)	-0.68 (-2.09 to 0.20)	< 0.001	-2.24 (-9.77 to -1.34)	-0.07 (-4.56 to 2.83)	0.007

Children with unresolved OSA (AHI ≥ 2 events/h or OAHI ≥ 1 event/h) at the trial endline were further included in the analysis

Change = Endline-Baseline

Z-score for weight-for-age change between baseline and the 7 months’ follow-up

## Discussion

The current study adds to existing literature that POSA status does not substantially influence the efficacy of AT in children with OSA, supporting AT as an effective treatment for pediatric OSA regardless of POSA status. However, the observed changes in POSA status over time in some individuals highlight the necessity for further in-depth research on OSA positional subtypes.

Our analysis of the CHAT cohort revealed a POSA prevalence of 47.2%, and this high prevalence aligned broadly with previous studies [9, 10, 12]. Children with POSA exhibited notably less severe OSA than their counterparts, possibly because airway obstruction primarily occurs in the supine position, allowing positional changes to reduce the AHI [7, 12]. Importantly, we found that children with POSA had longer REM sleep duration in the supine position and shorter REM sleep in the non-supine position, along with lower OAHI in both REM and NREM sleep. These observations suggest a potential interaction between sleep position and stage, which may influence the diagnosis of POSA, as respiratory events are generally more common during REM sleep. However, similar findings were not observed in a previous study focusing on obese children with and without POSA [10]. The identified correlations between sleep position, sleep stage, and OSA subtype highlight the complexity and the potential need to incorporate REM-specific criteria to refine POSA diagnoses in future studies and clinical management [14, 15]. Interestingly, we found a lower prevalence of allergies among children with POSA, though previous adult studies suggest that allergic diseases can exacerbate OSA by influencing neuromuscular control of pharyngeal muscles and causing sleep disruptions, the mechanism in children remains unclear [7].

The current study evaluated whether pediatric POSA status influences the effectiveness of AT, and our GLM models found that AHI reduction was significantly associated with randomization to the AT group but not with POSA status, and no interaction was found between these two factors across different panels of adjustments. Which provides evidence that POSA status generally does not significantly alter the effectiveness of AT. The RCS analysis further reinforces that AT consistently reduces AHI across patients with various baseline AHI levels irrespective of their POSA status. It demonstrates that AT treatment leads to more pronounced improvement in AHI among children with higher baseline AHI. Nonetheless, it is important to note that in this study population, more than half (54.2%) of the participants had mild OSA, and the majority (72.9%) of the children did not have very large tonsils. Moreover, previous studies have suggested that the exact location of the upper airway obstruction may also play an essential role in the development of POSA in children. *Kirkham EM* et al. found that children with POSA exhibited significantly more obstruction at the tongue base but not at other upper airway sites such as the adenoid, lateral wall, or supraglottis, as determined by drug-induced sleep endoscopy [13], this insight is crucial for surgical planning and preoperative counseling. Interestingly, *Ji Ho Choi* et al. identified the notable effect of AT on changes in sleep positions among children with OSA, finding that AT would significantly reduce positional changes during sleep and increase the proportion of children’s supine sleep as assessed by PSG [31]. Nonetheless, the mechanism needs further exploration.

Positional therapy (PT) aims to prevent patients from sleeping on their back, is considered as alternative therapy particularly for patients with POSA [7]. However, the majority of existing research focuses on adults, leaving its applicability in children uncertain. Importantly, we observed a dynamic transition of POSA status in approximately half of the children over time. In the WWSC group, 48.1% of children with POSA and 47.4% of children with non-POSA experienced alteration of their POSA classification at the 7-month follow-up. This finding suggests that the childhood POSA subtype may evolve over time. Nonetheless, further investigation is needed to elucidate whether these changes stem from limitations in the current commonly used classification methods which are adopted directly from adult studies, or reflect actual disease dynamics [19–21]. One retrospective study found that weight changes influence POSA diagnosis in adult, with weight gain linked to transitions from POSA to non-positional OSA and vice versa over an interval of 6.2 years [21]. Furthermore, in the adult study, the lateral AHI was a predictor of transitioning from POSA to a non-positional diagnosis [21]. Similarly, *Chou YT* et al. demonstrated that higher apnea index in the lateral position was the only independent indicator of changing from POSA to non-positional OSA in adults over 6 months to 2 years [20]. Our results align with these findings. In the WWSC arm, children’s transitions from POSA to non-POSA were associated with an increase in the non-supine OAHI over a 7-month follow-up. However, in the CHAT study, pre- and post-treatment PSG data were limited to single-night studies, raising concerns about the night-to-night variability (NtNV) and its impact on interpreting POSA dynamics. While earlier studies suggested limited NtNV in pediatric PSG [28], more recent studies have shown variability in OSA severity across nights [29, 30]. However, these studies often had small sample sizes or different methodologies, for example, using type 3 sleep monitors on older, overweight children, limiting their applicability to the CHAT cohort [29]. A recent review found no significant overall NtNV in AHI but significant variability in children with moderate-to-severe OSA. Given that 54.2% of our cohort had mild-to-moderate OSA, this variability warrants caution in interpreting dynamic change. To our knowledge, this is the first study to explore the natural progression of POSA in children. The identified variability of POSA over time highlights the need for regular follow-up assessments and adjustments in therapeutic interventions to optimize OSA management.

The CHAT study provides a robust dataset from a well-designed, rigorously conducted RCT evaluating multiple outcomes in otherwise healthy children with OSA. However, several limitations should be considered. First, the study primarily included children with mild-to-moderate OSA, excluding those needing urgent AT surgery or with severe comorbidities such as extreme obesity and poorly controlled allergies, consequently our findings may better represent children with less severe and less complex OSA. Second, the age range (5 to 9 years) while capturing the peak period of lymphoid tissue growth and symptom development, excludes younger and older children, limiting generalizability of current findings. Moreover, the relatively short follow-up might not fully capture the natural history of pediatric POSA. Importantly, the diagnostic criteria for pediatric POSA were extrapolated from adult classifications, commonly used in pediatric research due to the lack of pediatric-specific standards. Given children’s longer sleep durations and distinct sleep architecture, a global consensus of pediatric POSA diagnosis is needed. Furthermore, it remains unclear whether observed subtype transitions reflect true disease dynamics along with child development. The reliance on single-night PSG data for pre- and post-treatment assessments is a limitation, especially considering the first-night effect. Finally, the small sample size within transition subgroups restricted our ability to fully characterize disease progression. Larger cohorts and repeated PSG assessments are needed to refine our understanding of pediatric POSA.

## Conclusions

In summary, our findings demonstrate that AT is effective in managing pediatric OSA irrespective of POSA status. The observed instability of POSA status over time underscores the importance of regular monitoring when considering treatment alternatives such as positional device therapy. Future research should focus on refining the classification methods of POSA subtypes in children, exploring the natural course, treatment options and effects in this population to enhance personalized care and improve long-term outcomes.

## Electronic supplementary material

Below is the link to the electronic supplementary material.

Supplementary Material 1

## Data Availability

This analysis was made possible by the National Sleep Research Resource, with access to the CHAT database http://sleepdata.org/dataseets/chat (accessed on 7th Jun 2023).
