# Supplementary material for: Supine position-related obstructive sleep apnea in children: insights from the Childhood Adenotonsillectomy Trial
Source: Sleep Breath. 2025 Jun 30;29(4):230. doi: 10.1007/s11325-025-03393-1 (PMC12209019; doi:10.1007/s11325-025-03393-1)
Supplement: Supplementary file 1 — Supplementary Material 1 [file 11325_2025_3393_MOESM1_ESM.docx]

**Supplementary Table 1. AT effectiveness in outcome changes by POSA status and RCT grouping (N = 354)**

| Variables | POSA (N = 167) | | | | Non-POSA (N = 187) | | | | P^‡^ |
| --- | --- | --- | --- | --- | --- | --- | --- | --- | --- |
| Trial outcome changes | **All** | **Early AT (N = 80)** | **WWSC (N = 87)** | **P^*^** | **All** | **Early AT (N = 94)** | **WWSC (N = 93)** | **P**^†^ |  |
| AHI (no. of events/h), Median (IQR range) | -2.18 (-4.81 to -0.71) | -2.61 (-5.36 to -1.81) | -1.66 (-4.10 to 0.69) | 0.001 | -3.17 (-6.97 to -1.11) | -4.93 (-8.75 to -2.22) | -2.21 (-5.01 to 0.40) | < 0.001 | 0.03 |
| AHI supine (no. of events/h), Median (IQR range) | -5.18 (-10.38 to -2.24) | -6.03 (-14.32 to -3.21) | -3.63 (-8.79 to -0.67) | 0.003 | -2.34 (-7.02 to -0.04) | -3.46 (-8.71 to -1.66) | -1.18 (-4.51 to 3.25) | < 0.001 | < 0.001 |
| AHI non-supine (no. of events/h), Median (IQR range) | -0.58 (-1.49 to 0.44) | -0.96 (-1.89 to -0.20) | -0.36 (-1.00 to 1.89) | 0.002 | -3.25 (-6.89 to -1.34) | -5.02 (-8.78 to -2.61) | -1.75 (-5.03 to -0.33) | < 0.001 | < 0.001 |
| PSQ-SRBD score^&^ | -0.24 ± 0.03 | -0.28 ± 0.20 | -0.04 ± 0.18 | < 0.001 | -0.25 ± 0.03 | -0.27 ± 0.18 | -0.02 ± 0.19 | < 0.001 | 0.64 |
| NEPSY attention and executive-function score^#^ | 1.31 ± 2.41 | 7.00 ± 15.43 | 5.69 ± 13.93 | 0.59 | 4.15 ± 2.17 | 8.44 ± 13.37 | 4.29 ± 14.14 | 0.06 | 0.99 |
| Conners’ Rating Scale score^^^ |  |  |  |  |  |  |  |  |  |
| Caregiver rating | -3.18 ± 1.77 | -3.97 ± 12.32 | -0.80 ± 9.07 | 0.08 | -2.15 ± 1.49 | -2.00 ± 8.58 | 0.15 ± 10.12 | 0.15 | 0.23 |
| Teacher rating | -3.87 ± 2.98 | -4.71 ± 14.05 | -0.83 ± 11.93 | 0.20 | -3.42 ± 2.29 | -5.22 ± 12.57 | -1.80 ± 9.07 | 0.14 | 0.56 |
| BRIEF score^@^ |  |  |  |  |  |  |  |  |  |
| Caregiver rating | -4.55 ± 1.52 | -4.00 ± 9.34 | 0.55 ± 9.13 | 0.003 | -3.91 ± 1.31 | -3.13 ± 7.45 | 0.78 ± 8.94 | 0.003 | 0.62 |
| Teacher rating | -4.55 ± 2.73 | -3.92 ± 13.06 | 0.63 ± 10.88 | 0.10 | -2.20 ± 2.63 | -3.90 ± 11.45 | -1.69 ± 13.06 | 0.40 | 0.46 |
| PedsQL score^**^ | 2.75 ± 2.66 | 5.18 ± 14.32 | 2.43 ± 17.68 | 0.30 | -1.00 ± 2.56 | 2.37 ± 15.37 | 3.38 ± 16.49 | 0.69 | 0.64 |
| Persistent OSA, N (%) | 80 (47.9%) | 27 (33.8%) | 53 (60.9%) | 0.001 | 87 (46.5%) | 33 (35.1%) | 54 (58.1%) | 0.002 | 0.80 |

Data were presented as mean ± SD unless otherwise indicated

Changes of the outcome measures: Endline values at the 7-month’s follow up - Baseline values

^*^Group comparison between the EAT and WWSC children within the POSA children

^†^Group comparison between the EAT and WWSC children within the non-POSA children

^‡^Group comparison between the POSA and non-POSA group among the included children

Definition of abbreviations: POSA = positional obstructive sleep apnea; RCT = randomized controlled trial; AT = adenotonsillectomy; WWSC = watchful waiting plus supportive care; AHI = the apnea–hypopnea index; IQR = the interquartile range

^&^The Pediatric Sleep Questionnaire sleep-related breathing disorder scale (PSQ-SRBD) has scores that vary from 0 to 1, where higher scores reflect more severe symptoms

^#^In the attention and executive-function domain of the Developmental Neuropsychological Assessment (NEPSY), scores range from 50 to 150, with higher scores representing better cognitive functioning

^^^ Scores on the Conners’ Caregiver Rating Scale Revised: Long Version Global Index, which includes the Restless–Impulsive and Emotional Lability factor sets, span from 38 to 90, with higher scores denoting poorer functioning

The Conners’ Teacher Rating Scale Revised also measures similar aspects with scores ranging from 40 to 90, where higher scores indicate poorer functioning

^@^On the Behavior Rating Inventory of Executive Function (BRIEF) Global Executive Composite, which includes summary measures of behavioral regulation and metacognition, higher scores suggest worse executive functioning. Scores range from 28 to 101 for caregiver ratings and from 37 to 131 for teacher ratings

**The Pediatric Quality of Life Inventory (PedsQL) scores between 0 and 100, with higher scores indicating a better quality of life；Persistent OSA: post-study AHI ≥ 2 events/h
